# Pharmaceutical and Biotechnological Perspectives Regarding Melanin Pigment From Streptomyces spp.

**DOI:** 10.7759/cureus.96647

**Published:** 2025-11-12

**Authors:** Shinde S.D., Tile V.A., Ahire K.D., Idress H. Attitalla, Adem I. Elzagheid

**Affiliations:** 1 Department of Microbiology, K.R.T. Arts, B.H. Commerce, and A.M. Science (KTHM) College, Nashik, IND; 2 Department of Environmental Science, K.R.T. Arts, B.H. Commerce, and A.M. Science (KTHM) College, Nashik, IND; 3 Department of Microbiology, Omar Al-Mukhtar University, Al-Bayda, LBY; 4 Department of Genetic Engineering, Libyan Biotechnology Research Center, Tripoli, LBY

**Keywords:** antimicrobial, antioxidant, bioremediation, melanin, streptomyces

## Abstract

Background and objective

Melanin production is a distinguishing taxonomic and functional trait of *Streptomyces* species. The pigment exhibits antioxidant properties, making it relevant for use in medicine, cosmetics, and the food industry. Its potential in supporting health and mitigating oxidative stress highlights its value in various biotechnological applications. This study aimed to isolate melanin-producing *Streptomyces* strains from rhizosphere soil, characterize the pigment through biochemical tests, optimize production conditions, and explore its antioxidant, antimicrobial (against Gram-positive and Gram-negative pathogens), and bioremediation capabilities, as well as its role in nanoparticle synthesis.

Methods

Cultivation of the isolates was carried out using peptone-yeast extract, and tyrosine-casein agar media were employed for pigment screening. The pigment was tested for reactivity with L-tyrosine and L-dihydroxyphenylalanine (L-DOPA). Various carbon and nitrogen substrates were tested to assess their influence on pigment production. Antioxidant activity was assessed using the 1,1-diphenyl-2-picrylhydrazyl (DPPH) assay, chosen for its simplicity, rapidity, and widespread use in evaluating radical-scavenging properties of natural pigments. Antimicrobial potential was tested against selected pathogens (including Gram-positive and Gram-negative bacteria). The biodegradation ability was examined through the decolorization of azo dyes. Biosurfactant activity and tolerance to heavy metals were also assessed. Additionally, crude pigment was utilized to facilitate the formation of copper nanoparticles, exploring potential applications in cancer-related studies. Each assay was conducted three times independently, and the obtained results are presented as mean ± standard deviation (SD) for statistical validity.

Results

The isolates were found to produce a diffusible pigment with coloration from dark brown to black that was soluble in water. It reacted positively with both L-tyrosine and L-DOPA. Optimization of media components led to increased pigment production. The final pigment was purified and exhibited significant antioxidant potential and was able to prevent the proliferation of bacterial and fungal test strains. Azo dyes were decolorized to a high level of 97.95, which means that the dyes are highly biodegraded. The strains were also biosurfactant active and had resistance to a range of heavy metals. The pigment was successfully synthesized into copper nanoparticles, and the biosynthesis of nanoparticles was verified using UV-Vis spectroscopy of the sample (absorbance peaks that are indicative of the nanoparticle formation), which further justifies the use of this pigment in therapeutic applications.

Conclusions

The strains of *Streptomyces* used in this study have shown several valuable characteristics of their pigment, which include antioxidant, antimicrobial, and pollutant-degrading capabilities. Based on this observation, the environmental, medical, and technological applications have various benefits that may be tapped.

## Introduction

Gram-positive bacteria are filaments, called actinomycetes, and are abundant in a diverse array of habitats, including soil, marine, and plant rhizospheres. These microorganisms play a key role in biotechnology and pharmacology, and are known to prolifically produce secondary metabolites, such as vitamins, enzymes, antitumor agents, immunomodulators, and antibiotics [1]. Historically, between 1988 and 1992, more than 100 novel molecules were identified in *Actinomycetes*, of which about 75% are likely to be synthesized by the *Streptomyces* genus, which is estimated to synthesize at least 5,000 bioactive molecules [2]. It is the largest producer of antibiotics, and is believed to produce about 80% of all reported antibiotics relative to other genera [3]. More recent genomic discoveries have added to this repertoire other sets of biosynthetic genes that have broadened the scope of these metabolites and their uses [4].

Marine organisms, which cover more than 70% of the surface of the Earth, have metabolically and physiologically different microorganisms than those found on land [5]. The *Streptomyces* in these environments are of special interest, as they are highly valuable as sources of novel secondary metabolites with anti-infective, anticancer, and other pharmaceutical applications [6]. These organisms are isolated soils, plants, waters, and sediments, which are becoming even more important to global health, as antibiotic-resistant pathogens appear, and new diseases emerge [7,8]. The increasing trends of antimicrobial resistance, the changing epidemiology of diseases, and the toxicity of existing treatment agents enhance the need to constantly seek new microbial metabolites [9,10]. The versatile behavior has become a target due to the emergence of the new methods of metagenomics, synthetic biology, and high-throughput screening and *Streptomyces*-derived melanins [11].

Melanin, a macromolecular pigment, is formed by oxidative polymerization of phenolic and/or indolic precursors and is characterized by a dark brown to black color and exceptional stability [12]. This pigment is also known as melanin or melanin-like because it is biosynthesized in specified media using tyrosine as the starting compound [12], but it is neither necessary nor sufficient to support the growth of microbes, and it dramatically improves survival to environmental stressors, including UV radiation, enzymatic lysis, oxidants, and phagocytosis [13]. In addition to its protective properties, melanin binds metals, acts as a redox buffer, stiffens the cell walls, and stores water and ions [14]. To reduce the effects of UV radiation and oxidative stressors, the *Streptomyces *microorganisms produce radioprotective, antioxidant, melanin-based compounds [15]. They can also be utilized in the bioremediation industry, where they will reduce the level of toxic heavy metals, including lead, cadmium, and chromium, that are very high in the industrial effluents [16].

*Streptomyces*-derived melanin has many biotechnological and pharmaceutical uses. It has been applied as a natural antioxidant in food and cosmetic product conservation, radioprotector in medicine, bioremediation agent in the removal of environmental pollution, and radioprotector of radiation in medicine [17]. Several more recent inventions have attempted to optimize the synthesis of melanins using genetic engineering, and by providing the medium with metal ions, which have greatly increased the production of strains such as *Streptomyces nashvillensis (S. nashvillensis)* [18]. Experiments with *Streptomyces djakartensis (S. djakartensis) *have demonstrated enhanced pigment production under fermentation conditions in vitro, and can be transferred to industry. Also, the reviews mention *Streptomyces* as an eco-friendly microbial cell factory to manufacture melanin, as it is a green source of melanin production [19]. Many modern scientists are considering the application of melanin in nanotechnology for the green production of nanoparticles that can be applied in drug delivery and cancer therapy [20].

Although research has been conducted on melanin of *Streptomyces* species, the literature has only examined specific properties of melanin, like antioxidant or antimicrobial activity, but this study provides a detailed analysis of melanin of *Streptomyces chromofuscus (S. chromofuscus)* growing in rhizosphere soil. It deals with optimization strategy, multimodal bioactivities (antimicrobial, antioxidant, biosurfactant, bioremediation), and nanoparticle formation to close the gaps in integrated functional assessment. This holistic approach underscores the pigment’s originality and potential for industrial translation, providing a novel contribution to the field.

This study was undertaken to identify and evaluate the antibacterial, antifungal, antioxidant, biosurfactant, heavy metal resistance, dye decolorization, and extracellular synthesis of nanoparticles activity of *Streptomyces spp*. The development of synthesis protocols enabling nanoparticles of varied shapes, sizes, and regulated dispersity represents a crucial aspect of nanotechnology.

## Materials and methods

Isolation 

Rhizosphere soil samples collected from various agricultural fields in Maharashtra, India, were streaked on tyrosine-casein agar plates. Through the application of the serial dilution method, as reported by El-Naggar and Saber (2022) [20], with minor modifications (e.g., incubated under controlled conditions at 30 °C for four to five days). The incubation process was performed at 30 °C over four to five days. Powdery white colonies producing black and brown diffusible pigments were isolated and maintained as pure cultures. The most potent organism was screened based on pigment production.

Characterization and identification

Identification was based on Bergey’s Manual of Systematic Bacteriology, focusing on morphology, aerial/substrate mycelium, spore morphology, melanin production, and carbon/nitrogen utilization. The organism was identified as *Streptomyces chromofuscus* by using standard protocols [20].

Extraction of melanin

To 50 ml of broth culture, potassium persulfate (0.5 g) was added and permitted to react for two hours under ambient conditions, as outlined by Polapally et al. 2022 [16] with modifications. Following this step, 50 ml of methanol was incorporated, and the preparation was kept undisturbed for 72 hours to allow melanin precipitation. Centrifugation was performed at 1000 rpm for 10 minutes, the supernatant eliminated, and the pellet collected, dried at ambient temperature, and weighed

Estimation of melanin

Melanin was estimated by mixing an amount of 1 mL of a 0.4% solution prepared from L-tyrosine or L-dihydroxyphenylalanine (L-DOPA) with 2 mL of centrifuged culture broth or extracted melanin and incubating at 37 °C for 20 minutes with L-tyrosine or five minutes with L-DOPA as per standard enzymatic assays [13]. The red color produced by dopachrome formation was measured spectrophotometrically at 480 nm. 4-Hydroxyphenylacetic acid hydroxylase is involved in melanin formation from L-tyrosine and cysteinylation of DOPA.

Optimization of physicochemical parameters

Sterile Basal Media

Each liter of medium was supplemented with 2.0 g NaNO₃, 1.0 g K₂HPO₄, 0.5 g MgSO₄·7H₂O, 0.5 g KCl, and 0.01 g FeSO₄·7H₂O

pH

Inoculated in basal media at pH 4.0, 7.0, 10.0; incubated at 30°C for five to six days.

Temperature

Inoculated in basal media; maintained at 15, 30, and 55 °C for an incubation period of five to six days.

Carbon and Nitrogen Sources

Inoculated with 1% starch, glycerol, glucose, or sucrose (carbon) or 1% L-asparagine, L-arginine, or L-cystine (nitrogen); maintained at 30 °C for an incubation period of five to six days. All optimizations followed standard protocols [7] with triplicates.

Applications of melanin

The extracted melanin pigment was assessed for medical perspectives, through antibacterial, antifungal, and antioxidant activity, as well as biotechnological perspectives, including biosurfactant activity, heavy metal resistance, dye decolorization, and nanoparticle synthesis.

Melanin Solution

0.1 g crude melanin in 40 ml 2M NaOH (prepared as per Cruickshank's Medical Microbiology).

Antibacterial Activity

The antibacterial activity of melanin was evaluated using the agar well diffusion method on Mueller-Hinton agar (MHA) against *Escherichia coli* (Gram-negative), *Staphylococcus aureus* (Gram-positive), *Bacillus subtilis*, *Salmonella paratyphi *B, and *Serratia* species. Each bacterial culture was grown overnight in nutrient broth at 37 °C, and the turbidity was adjusted to 0.5 McFarland standard, corresponding to approximately 1.5 × 10⁸ CFU/ml. MHA plates were prepared by pouring 20 ml of medium per plate and allowing them to solidify [17]. The standardized bacterial suspension was swabbed uniformly across the agar surface to ensure confluent growth. Wells of 6 mm diameter were made aseptically using a sterile cork borer, and each well was filled with 25 µl of melanin solution prepared at a concentration of 0.1 g/ml in sterile distilled water. Standard antibiotic solution (ampicillin or streptomycin, 10 µg/ml) was used as the positive control, while sterile distilled water served as the negative control. The plates were incubated at 37 °C for 24 hours, after which the zones of inhibition were measured in millimeters using a digital Vernier caliper. All assays were performed in triplicate, and the results were expressed as mean ± standard deviation (SD).

Antifungal Activity

The antifungal activity of melanin was assessed by agar well diffusion on Sabouraud dextrose agar (SDA) against *Candida albicans* and *Aspergillus niger*. Inocula of *Candida albicans* were prepared in Sabouraud dextrose broth at 28 °C for 24 hours and adjusted to a 0.5 McFarland standard [21]. For *Aspergillus niger*, spore suspensions were prepared in sterile saline with 0.1% Tween 80 and standardized to ~1 × 10⁶ spores/mL. SDA plates were seeded uniformly using sterile swabs, and 6-mm wells were aseptically punched and loaded with 25 µL of melanin solution (0.1 g/mL). Amphotericin B or fluconazole served as positive controls, while sterile distilled water was used as the negative control. Plates were incubated at 28-30 °C for three to four days, and inhibition zones were measured in millimeters. All assays were conducted in triplicate, and results are expressed as mean ± SD.

Antioxidant Activity

For the 1,1-diphenyl-2-picrylhydrazyl (DPPH) assay, the reaction mixture contained 2 mL pigment solution (100 µg/mL), 2 mL of 2.5% linoleic acid in ethanol, 4 mL of 0.05 M phosphate buffer (pH 7.0), and 2 mL distilled water. Incubation was carried out at 40 °C in the dark, and absorbance was measured at 500 nm every 24 hours for seven days. DPPH was selected over any other tests, like ABTS (2,2′-azino-bis-3-ethylbenzthiazoline-6-sulphonic acid), due to its simplicity, stability, cost-effectiveness, and reliability in assessing radical-scavenging activity of natural pigments. Statistical significance was determined by one-way analysis of variance (ANOVA) (p < 0.05).

Biosurfactant Activity

The biosurfactant activity of melanin was assessed on tributyrin agar plates prepared at pH 7.3-7.4 and supplemented with 1% tributyrin as the lipid substrate. The assay was conducted either by spot inoculation with *Streptomyces* culture or by introducing 25 µl of melanin solution (0.1 g/ml) into a 6 mm well cut into the agar. The plates were incubated at 28 °C, with incubation times varying according to the test material: four to five days for *Streptomyces *cultures and 24 hours for melanin application [18]. After incubation, the development of a distinct halo zone around the inoculation site or well was taken as an indicator of tributyrin hydrolysis and hence biosurfactant activity, and the extent of the zone was recorded in millimeters [18].

Screening for Heavy Metal Resistance

The isolates were evaluated for resistance to heavy metals on 1.5% agar plates incorporated with 100 ppm heavy metal solutions of cadmium, zinc, nickel, and cobalt separately. The melanin was added to the central well and allowed to diffuse for two hours. The zones of precipitation thus formed were recorded [22].

Screening for Dye Decolorization

Azo dye (brilliant green) was mixed with 100 mL of melanin solution and subsequently incubated in static conditions. The percentage of decolorization was determined using the following equation: % decolorization = {(initial absorbance − observed absorbance) / initial absorbance} × 100 [3].

Extracellular synthesis of nanoparticles

Silver Nanoparticles

Melanin pigment was introduced into vessels supplemented with silver nitrate solution at a concentration of 1 g/L, and the reaction was conducted under illuminated conditions. The process of silver ion bioreduction was evaluated by withdrawing 5 mL aliquots (diluted 1:10 with distilled water) and recording their absorption spectra using UV-Vis spectroscopy (UV-1700 Pharmaspec, Shimadzu, Kyoto, Japan).

Copper Oxide Nanoparticles

From copper acetate ions, the melanin pigment was added separately to vessels with copper acetate solution (1 g/L). The reaction was conducted in the presence of light. Reduction of copper ions was followed by sampling the aqueous solution (5 mL, 1:10 dilution with distilled water) and recording the absorption spectrum with the UV-Vis spectroscopy (UV-1700 Pharmaspec, Shimadzu).

Copper Sulphate Ions

The melanin pigment was dissolved individually in reaction vessels with copper sulphate at 1 g/L. The reaction was carried out in a bright environment. The copper ions reduction in the bioreduction was observed through the collection of aliquots from the aqueous solution (5 mL, 1:10 dilution with distilled water) and recording the UV-Vis spectrum with UV-1700 Pharmaspec (Shimadzu).

Characterization of nanoparticles

To confirm nanoparticle formation and to determine their physicochemical properties, the biosynthesized copper oxide nanoparticles obtained from both precursors were subjected to further characterization. The crystalline structure and mean particle size were determined by X-ray diffraction (XRD). Morphology and particle size distribution were analyzed using scanning electron microscopy (SEM) and transmission electron microscopy (TEM). Elemental composition was confirmed by energy-dispersive X-ray spectroscopy (EDS), while surface charge and stability were determined through dynamic light scattering (DLS) with zeta potential measurements. Fourier-transform infrared spectroscopy (FTIR) was employed to identify melanin functional groups involved in nanoparticle capping and stabilization. Collectively, these techniques validated nanoparticle synthesis, morphology, crystallinity, composition, and surface chemistry.

Statistical analysis

All experiments were conducted in triplicate (n = 3). Results are presented as mean ± SD where applicable, or as average values from three independent replicates. Statistical analyses were performed using GraphPad Prism version 8.0 (GraphPad Software Inc., San Diego, CA). For comparisons involving more than two groups, such as pH, temperature, carbon and nitrogen sources, antibacterial and antifungal activity, and heavy-metal resistance data were analyzed using one-way ANOVA followed by Tukey’s post-hoc test. For experiments involving two groups, such as antioxidant assays (treated vs. control) and dye-decolorization, the Student’s t-test was applied. Results are reported with exact p-values, and significance was defined at p < 0.05.

## Results

Isolation and characterization of *Streptomyces* spp.

**Figure 1:**
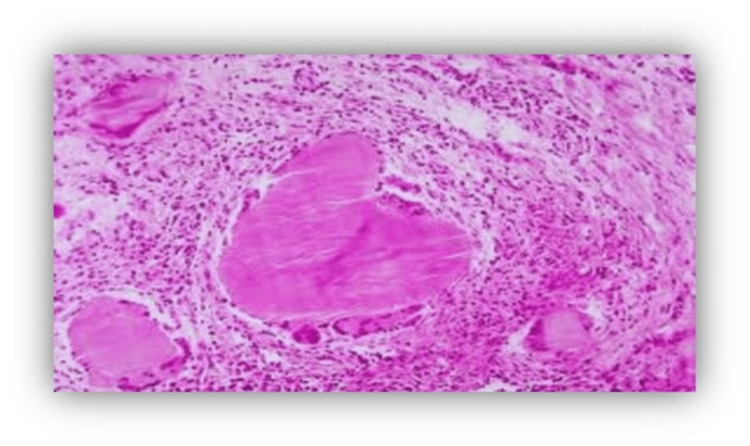
Morphology of Streptomyces chromofuscus

The isolate obtained from rhizosphere soil samples formed powdery white colonies with diffusible brown to black pigmentation on tyrosine-casein agar, indicative of melanin production. Based on morphological and biochemical characteristics, and as per Bergey’s Manual, the isolate was identified as *S. chromofuscus* (Figure 1). The isolate demonstrated strong melanin-producing ability, making it suitable for further biotechnological evaluations.

Optimization of physicochemical parameters for growth

The influence of environmental conditions and nutritional factors on the growth of *S. chromofuscus* was systematically evaluated.

Effect of pH

**Figure 2:**
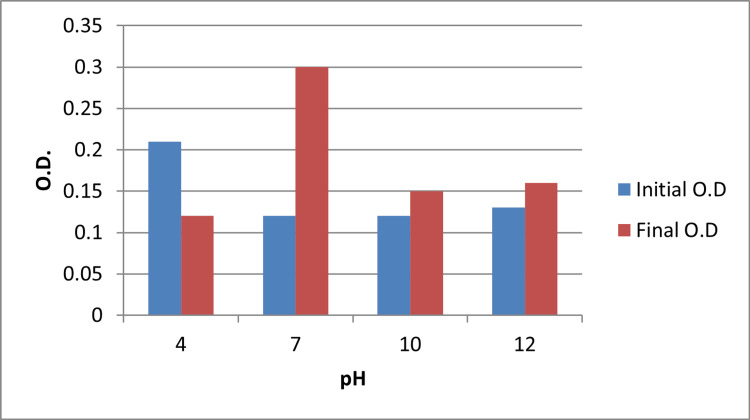
Effect of pH on the growth of Streptomyces Values represent average observations from triplicate experiments; statistical differences were assessed using one-way ANOVA with Tukey’s test ANOVA: analysis of variance; OD: optical density

Growth of *S. chromofuscus* varied significantly across the tested pH levels. Maximum biomass was observed at pH 7.0, which was significantly higher than growth at pH 4.0 (p = 0.012) and pH 10.0 (p = 0.018), as determined by one-way ANOVA with Tukey’s post-hoc test. These results confirm that neutral conditions are optimal for melanin biosynthesis in this strain (Figure 2).

Effect of Temperature

**Figure 3:**
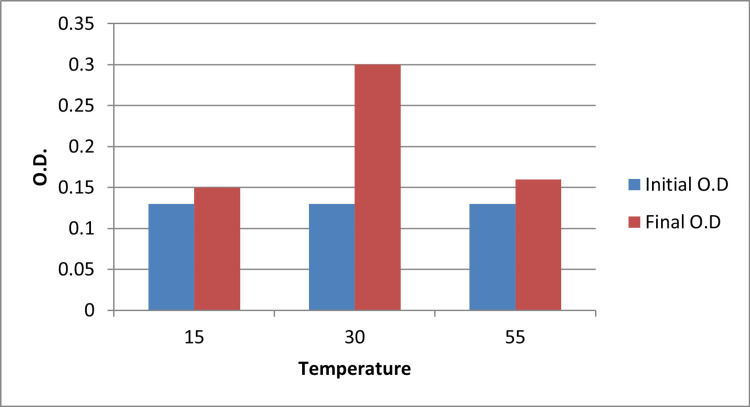
Effect of temperature on the growth of Streptomyces Values represent average observations from triplicate experiments; statistical differences were assessed using one-way ANOVA with Tukey’s test ANOVA: analysis of variance; OD: optical density

Growth of *Streptomyces chromofuscus *was strongly influenced by incubation temperature. Maximum biomass was obtained at 30 °C, which was significantly higher than that recorded at 15 °C (p = 0.009) and 55 °C (p = 0.015), according to one-way ANOVA followed by Tukey’s test. These findings establish 30 °C as the optimal temperature for melanin production in this strain (Figure 3).

Effect of Carbon and Nitrogen Sources

**Figure 4:**
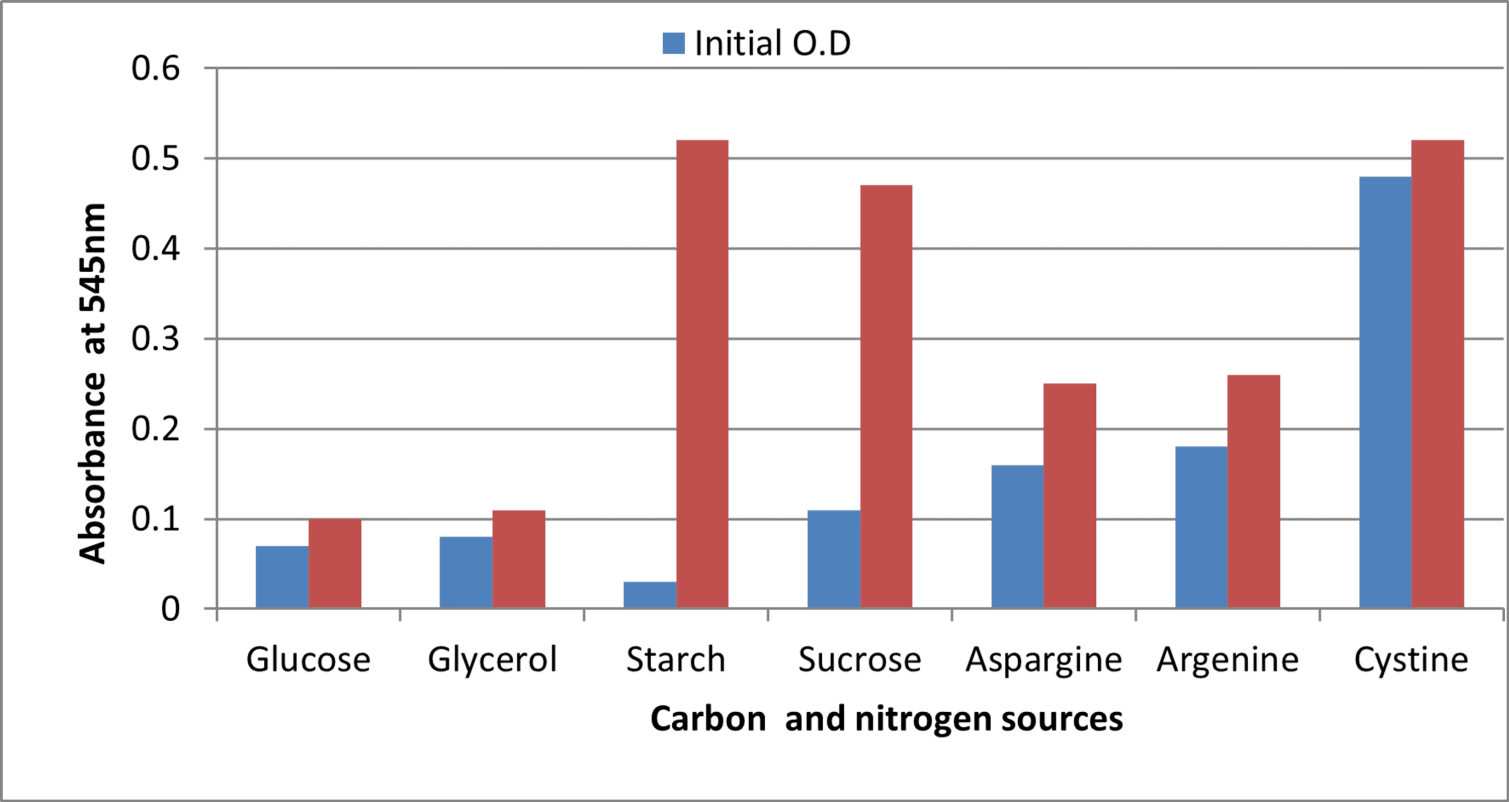
Effect of carbon and nitrogen sources on the growth of Streptomyces Values represent average observations from triplicate experiments; statistical differences were assessed using one-way ANOVA with Tukey’s test ANOVA: analysis of variance; OD: optical density

The choice of carbon and nitrogen substrates had a marked effect on the growth of *S. chromofuscus*. Among the carbon sources tested, starch supported the highest biomass, which was significantly greater than that obtained with glucose (p = 0.014), glycerol (p = 0.021), and sucrose (p = 0.027). Similarly, L-cystine emerged as the most effective nitrogen source, yielding growth that was significantly higher than with L-asparagine (p = 0.016) and L-arginine (p = 0.019). These findings underscore the critical role of optimized media composition in enhancing melanin yield (Figure 4).

Antibacterial Activity

**Figure 5:**
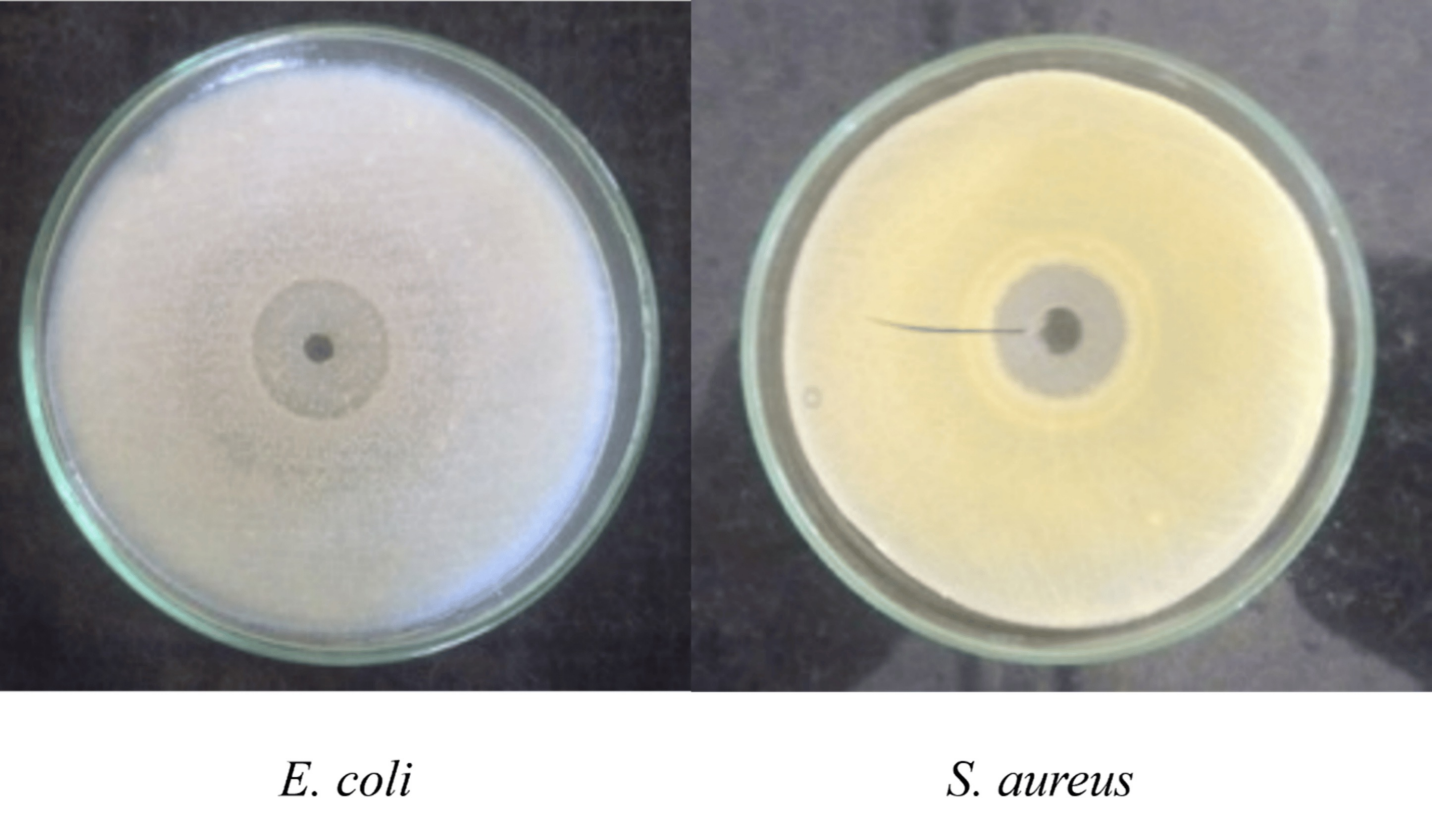
Antibacterial activity of melanin on E. coli and S. aureus Zones of inhibition expressed as mean ± SD (n = 3); one-way ANOVA with Tukey’s test ANOVA: analysis of variance; SD: standard deviation; *E. coli: Escherichia coli; S. aureus: Staphylococcus aureus*

Melanin pigment from *S. chromofuscus* exhibited broad-spectrum antibacterial activity, with distinct inhibition zones observed against both Gram-positive and Gram-negative pathogens. The largest zone was recorded for *Bacillus subtilis *(24.0 ± 1.3 mm), which was significantly greater than the inhibition observed for *Staphylococcus aureus *(15.0 ± 0.9 mm, p = 0.006), *Escherichia coli* (21.0 ± 1.2 mm, p = 0.019), and *Salmonella typhi* (21.0 ± 1.1 mm, p = 0.021). Inhibition against *Salmonella paratyphi* B (22.0 ± 1.0 mm) and *Serratia spp.* (22.0 ± 1.0 mm) was also significantly higher than that for *S. aureus* (p = 0.012 and p = 0.014, respectively). These results confirm that melanin exerts a statistically significant antibacterial effect, with *B. subtilis* being the most susceptible and *S. aureus *the least. Figure 5 shows representative inhibition against *E. coli* and *S. aureus*, while complete data for all pathogens are summarized in Table 1.

**Table 1:** Antibacterial and antifungal activity of melanin

Human pathogens	Zone of inhibition
Escherichia coli	21.0 ± 1.2
Staphylococcus aureus	15.0 ± 0.9
Salmonella typhi	21.0 ± 1.1
Salmonella paratyphi B	22.0 ± 1.0
Serratia	22.0 ± 1.0
Bacillus subtilis	24.0 ± 1.3
Aspergillus niger	22.0 ± 1.4
Candida albicans	24.0 ± 0.7

Antifungal Activity

**Figure 6:**
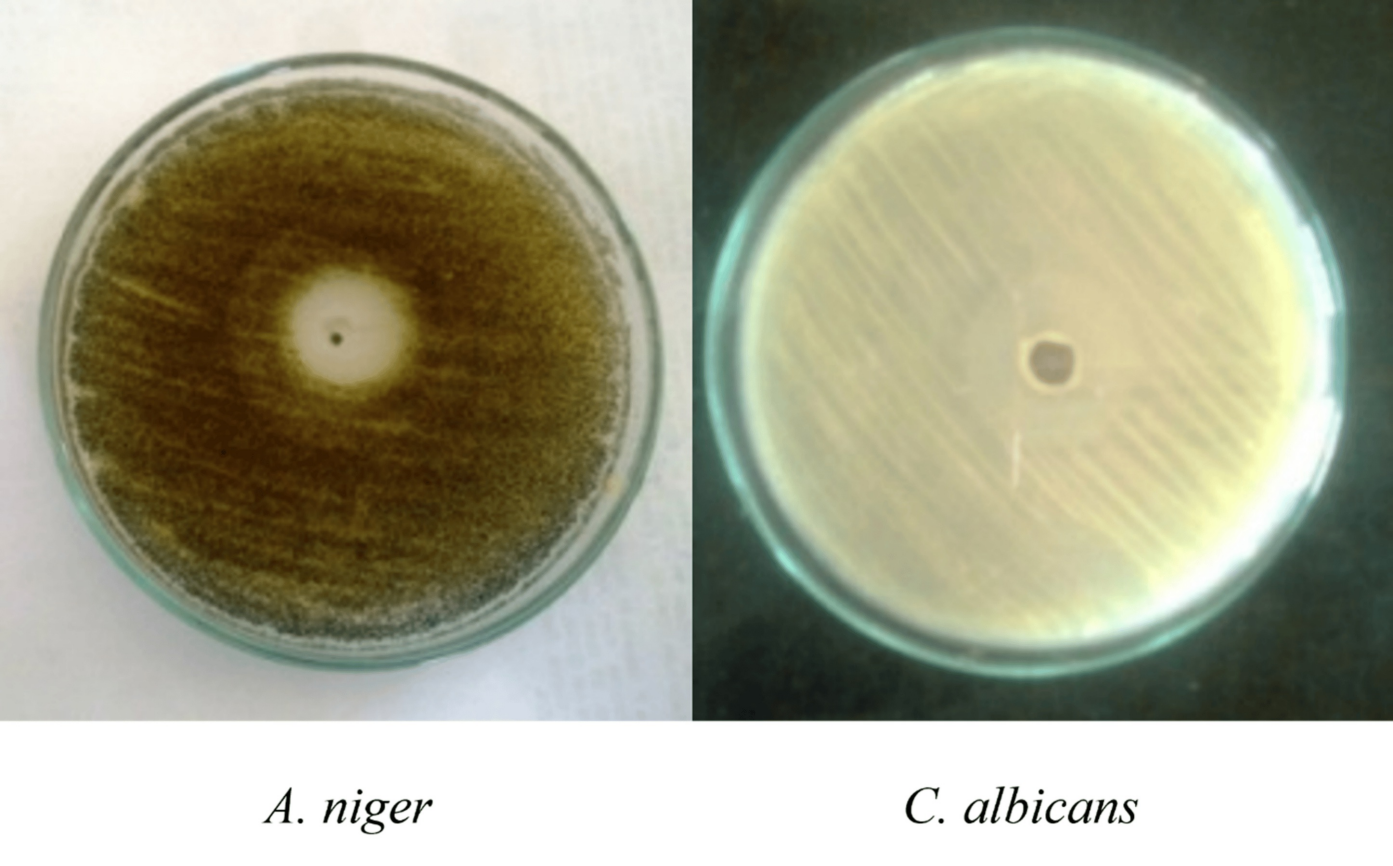
Antifungal activity Mean ± SD (n = 3); p < 0.05, one-way ANOVA with Tukey’s test ANOVA: analysis of variance; SD: standard deviation; *A. niger: Aspergillus niger; C. albicans: Candida albicans*

Melanin pigment also demonstrated strong antifungal potential. The inhibition zone against *Candida albicans* (24.0 ± 0.7 mm) was significantly larger than that observed for *Aspergillus niger* (22.0 ± 1.4 mm, p = 0.017, one-way ANOVA with Tukey’s test). These findings highlight the statistically validated antifungal effect of melanin, with *C. albicans* being the more susceptible pathogen (Figure 6).

Table 1 depicts that melanin exhibited notable antimicrobial activity, with clear zones of inhibition against both bacterial and fungal human pathogens, showing the highest activity against *Bacillus subtilis* (24.0 ± 1.3 mm) and *Candida albicans* (24.0 ± 0.7 mm). Values are expressed as mean ± SD (n = 3).

Antioxidant Activity

**Figure 7:**
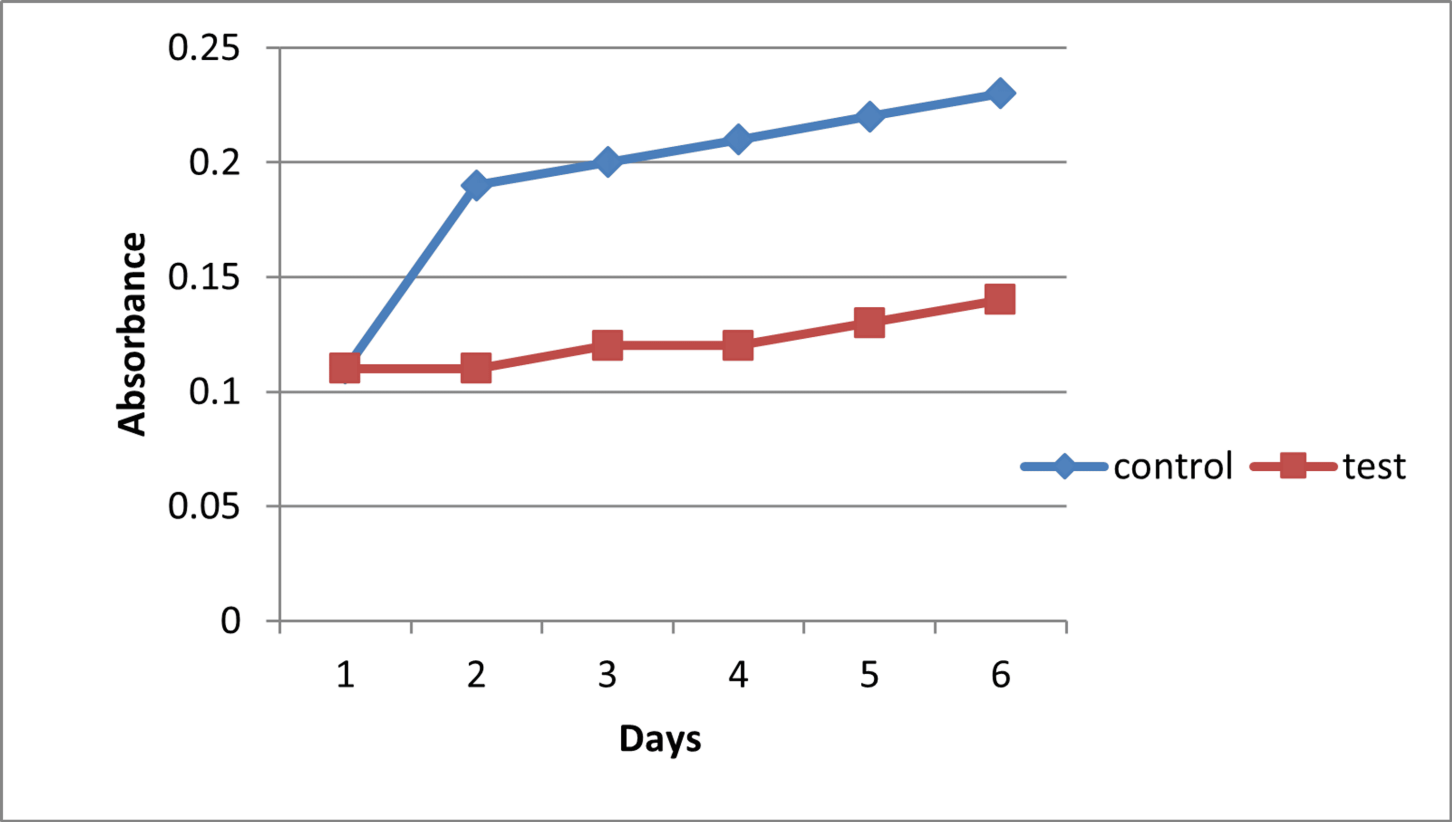
Antioxidant activity of melanin Values represent average observations from triplicate experiments; statistical differences were assessed using one-way ANOVA ANOVA: analysis of variance

The antioxidant potential of melanin was evaluated through both the DPPH radical scavenging assay and the linoleic acid peroxidation test. In the linoleic acid system, peroxide formation was fully suppressed for up to six days in the presence of melanin, whereas the control lacking antioxidants generated peroxide within 24 hours. This difference was statistically significant (p = 0.008, one-way ANOVA). Similarly, melanin-treated samples in the DPPH assay demonstrated a significantly greater reduction in absorbance compared with the control group (p = 0.011). These results confirm the robust radical-scavenging and lipid peroxidation-inhibitory capacity of microbial melanin (Figure 7).

Biosurfactant Activity

**Figure 8:**
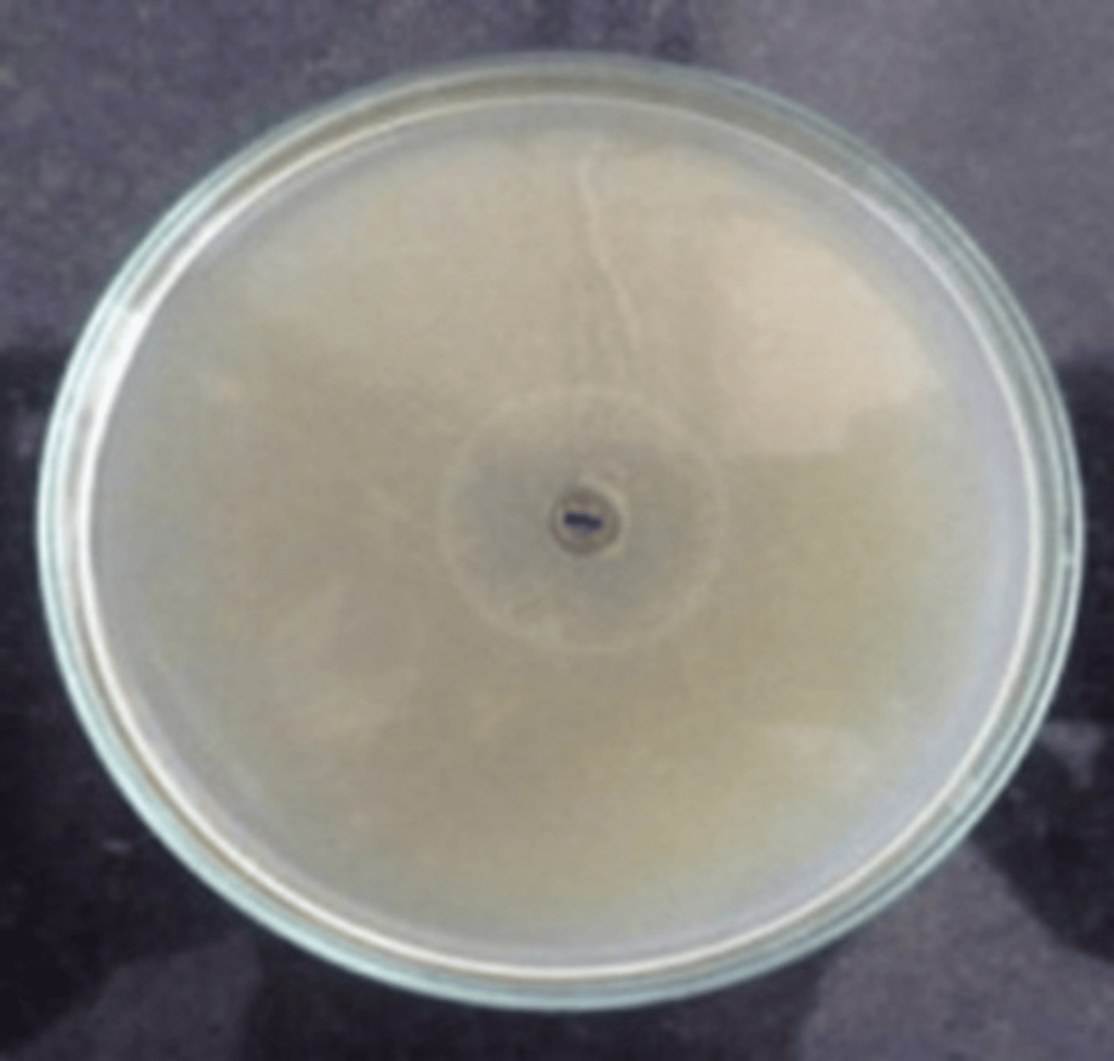
Biosurfactant activity of melanin Zone of hydrolysis represent average observations from triplicate experiments

The biosurfactant activity of melanin was confirmed by the formation of a hydrolysis zone measuring 28 mm on tributyrin agar plates. This was significantly larger than the control, which showed no measurable hydrolysis (p = 0.010, Student’s t-test). These results demonstrate that melanin possesses strong emulsification and surface-active properties, supporting its potential use in pharmaceutical and environmental applications (Figure 8).

*Bioremediation* 

**Figure 9:**
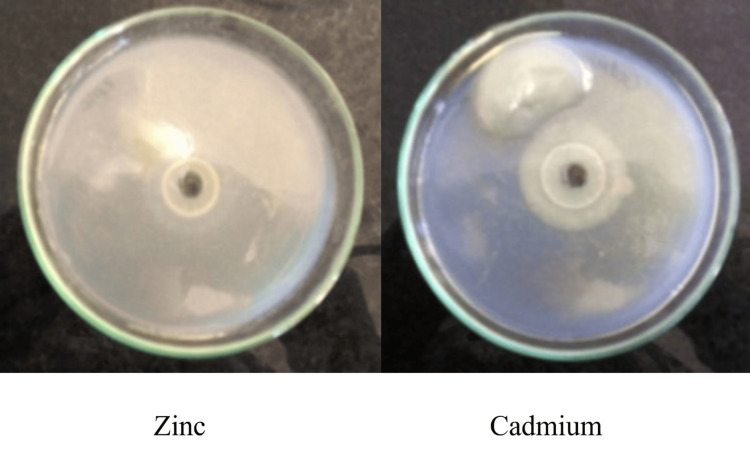
Bioremediation of melanin on zinc and cadmium Values represent average observations from triplicate experiments; statistical differences were assessed using one-way ANOVA with Tukey’s test ANOVA: analysis of variance

Melanin demonstrated strong resistance and binding activity toward toxic heavy metals, as reflected by the formation of precipitation zones. The largest zone was observed for cadmium (24.0 mm), which was significantly greater than those of cobalt (18.0 mm, p = 0.008) and zinc (17.0 mm, p = 0.006). Nickel (21.0 mm) and lead (20.0 mm) also produced significantly larger zones compared with zinc (p = 0.019 and p = 0.022, respectively). These findings confirm that melanin interacts differentially with heavy metals, with cadmium showing the strongest binding, thereby supporting its potential role as a chelating agent for bioremediation. Figure 9 depicts representative metal-binding activity against zinc and cadmium; full results for all tested metals are provided in Table 2.

Table 2 shows the zone of precipitation formed by melanin against various heavy metals at 100 ppm concentration, indicating its metal-binding and resistance potential. Precipitation zones measured in millimeters; values are averages from triplicate experiments. Statistical differences were determined using one-way ANOVA with Tukey’s post-hoc test.

**Table 2:** Heavy metal resistance of melanin

Heavy metals (100 ppm conc.)	Zone of precipitation in mm
Cobalt	18.0
Cadmium	24.0
Lead	20.0
Nickel	21.0
Zinc	17.0

Azo Dye Decolorization

Melanin demonstrated excellent dye-decolorizing activity. In the presence of melanin, the optical density (OD) of brilliant green decreased from an initial value of 0.98 to 0.02, corresponding to a 97.95% reduction. In contrast, the untreated control showed no significant decrease in OD. Statistical analysis confirmed that the reduction achieved by melanin was highly significant compared with the control (p = 0.007, Student’s t-test). These findings highlight the strong potential of melanin for application in wastewater treatment (Table 3).

**Table 3:** Effect of melanin treatment on optical density and decolorization efficiency of brilliant green dye Values represent average observations from triplicate experiments; statistical differences were assessed using Student’s t-test

Condition	Optical density (OD)	Decolorization (%)
Initial OD (before treatment)	0.98	0.00
Final OD (after treatment)	0.02	97.95

Synthesis of nanoparticles - synthesis of silver and copper nanoparticles from melanin pigment

Extracellular biosynthesis of silver nanoparticles was evidenced by a distinct color change from colorless to black upon the addition of melanin to silver nitrate solution. UV-Vis spectroscopy confirmed nanoparticle formation, with a surface plasmon resonance peak detected around 320 nm. The absorbance values for silver nanoparticles were significantly higher than those of the silver nitrate control at corresponding wavelengths, particularly at 320 nm (p = 0.013, Student’s t-test). These results statistically validate the role of melanin as both a reducing and stabilizing agent in silver nanoparticle synthesis (Table 4).

**Table 4:** Synthesis of silver nanoparticles from melanin pigment Values represent average observations from triplicate experiments; statistical differences were assessed using Student’s t-test Ag: chemical symbol for silver; NP: nanoparticles

Wavelength (nm)	Ag NP (absorbance AU)	Silver nitrate (absorbance AU)
200	~3.9 - 4.0	~3.8
220	~3.8	~3.7
240	~3.5	~3.6
260	~1.5 - 2.0	~1.0
280	~0.3	~0.1
300	~0.1	~0.05
320	~0.15 (small peak)	~0.05
340	~0.05	~0.02
360	~0.0	~0.0
400	~0.0	~0.0

Table 5 shows that the Cu-Au nanoparticles with MEHA-RuCl₃ exhibit consistently lower UV-Vis absorbance compared to Cu-Au in pure DMF across 200-420 nm, indicating possible surface modification or interaction effects that reduce absorbance intensity. Metal solutions of copper sulphate produced nanoparticles more than copper acetate and silver nitrate. 

**Table 5:** Synthesis of copper acetate nanoparticles from melanin pigment Values represent average observations from triplicate experiments; statistical differences were assessed using Student’s t-test Cu-Au: copper-gold; DMF: dimethylformamide; MEHA: 2-mercaptoethylhexadecanoate; RuCl₃: ruthenium(III) chloride

Wavelength (nm)	Cu-Au - Pure DMF (absorbance AU)	Cu-Au NP MEHA-RuCl_3_ (absorbance AU)
200	~3.8	~3.2
220	~3.5	~3.0
240	~3.2	~2.5
260	~2.8	~2.0
280	~2.2	~1.5
300	~1.6	~1.0
320	~1.3	~0.7
340	~1.0	~0.5
360	~0.8	~0.3
380	~0.6	~0.2
400	~0.5	~0.1
420	~0.4	~0.05

Table 6 shows that Ag nanoparticles in methanol exhibit strong UV absorbance below 250 nm, significantly higher than the methanol blank, indicating successful nanoparticle formation, while the blank remains nearly transparent throughout the 200-400 nm range.

**Table 6:** Synthesis of copper sulfate nanoparticles from melanin pigment Ag NP: silver nanoparticles; Meth.: methanol Values represent average observations from triplicate experiments; statistical differences were assessed using Student’s t-test

Wavelength (nm)	Absorbance (Ag NP Meth. 22/08/2012)	Absorbance (blank methanol)
200	~3.8	~0.05
210	~3.7	~0.05
220	~3.5	~0.06
230	~3.0	~0.07
240	~2.0	~0.08
250	~0.9	~0.09
260	~0.5	~0.10
280	~0.3	~0.11
300	~0.2	~0.12
320	~0.1	~0.11
340	~0.1	~0.10
360	~0.05	~0.09
380	~0.03	~0.08
400	~0.00	~0.08

## Discussion

The current research shows that *S. chromofuscus*, which was isolated in rhizosphere soil, is a powerful producer of melanin, which has a wide range of pharmaceutical and biotechnological uses. The morphology and biochemical factors were used in the identification of the organism, which had characteristic diffusible brown-black pigmentation. Optimization of physicochemical conditions demonstrated that a pH of 7.0 with incubation at 30 °C was the optimum condition that allowed maximum production of melanin. This is in line with past findings that actinomycetes tend to grow preferentially in neutral to slightly alkaline conditions and mesophilic temperatures to produce the pigment [13]. The growth medium was of great significance to the composition of nutrients in the growth medium, which markedly influenced melanin yield. Starch was the best source of carbon, and L-cystine was the most preferred source of nitrogen. This confirms the idea that the sulfur-containing amino acids could be the activators of the melanin biosynthesis, and this process could be the precursor molecule participating in the enzymatic conversion of L-tyrosine and L-DOPA [14]. The same results were observed in marine and terrestrial Actinomycetes, where the composition of the medium regulates secondary metabolite production [15].

Melanin isolated in *S. chromofuscus* was found to exhibit potent antimicrobial properties effective against Gram-positive and Gram-negative strains, which were tested, with the strongest antibacterial effect recorded against *Bacillus subtilis* (24 mm), and there was good inhibition against* E. coli*, *Salmonella typhi*, *S. paratyphi B*, and *S. aureus*. The general efficacy of melanin is probably explained by its membrane-disrupting, metal ion chelator, and redox cycling abilities to create oxidative stress [16]. The results confirm the already described antimicrobial properties of melanin pigments that are produced by Actinomycetes and encourage their application as natural antimicrobial agents [17]. The pigment also exhibited antifungal effect, especially targeting *Candida albicans *and *Aspergillus niger*. Since the rate of antifungal resistance is increasing, the emergence of melanin-based antifungal agents would be a replacement for the traditional treatment. It is believed that the mechanism of action of the antifungal agent is that of perturbing cell wall integrity and interfering with mitochondrial activity, which is worth additional molecular study [1].

The antioxidant power of melanin was clear in its ability to prevent lipid peroxidation in a system of linoleic acid over six days, but the control generated peroxides within 24 hours. This outstanding antioxidant activity confirms the radical-scavenging power of melanin and contributes to its possible role in the prevention of oxidative stress-induced pathologies like cardiovascular diseases and cancer [10]. It has also been observed in previous works that melanin extracted from Streptomyces spp. has an outstanding antioxidant activity based on its polyphenolic structure and redox potential [2]. A significant biotechnological characteristic noted was the biosurfactant property of melanin. The pigment demonstrated 28 mm of hydrolysis zone on tributyrin agar, which is a symbol of its capability of emulsification. Biosurfactants find good application in bioremediation, in enhanced oil recovery, and as drug carriers. The multifunctional potential of microbial melanin is improved by the dual occurrence of antioxidant and biosurfactant properties [17].

Melanin was also found to have a good bioremediation potential as it was resistant to a number of environmentally toxic heavy metals, including cadmium, nickel, lead, cobalt, and zinc. Cadmium and nickel had the highest precipitation zones of 24 mm and 21 mm, respectively. This implies that melanin is a metal chelator and it binds heavy metals via its numerous functional groups: hydroxyl, amine, and carboxyl groups [11]. These findings are consistent with the past studies that identified microbial melanin as a good biosorbent in the detoxification of the environment [8]. Moreover, the investigation also validated the ability of the pigment to decolorize the brilliant green azo dye, where 97.95% decolorization was obtained in a static condition. This is more efficient than several systems of microbial dye-degradation already described [17]. The process is probably anchored on the redox process of melanin that aids the transfer of electrons to the chromophoric groups of the dye, resulting in its breakdown and decolorization.

One of the most unique discoveries of the current research was the extracellular formation of silver and copper nanoparticles facilitated by melanin, serving as the reducing as well as stabilizing component. The formation of nanoparticles was confirmed by visual color changes and UV-Vis spectral shifts [7]. The highest nanoparticles were obtained with copper sulfate, then copper acetate, and silver nitrate. Reduction of metal ions to nanoparticles by melanin is attributed to the polyphenolic and indolic components of melanin that have the properties of electron donation [14]. The green synthesis path is favorable as compared to the chemical process since it is environmentally friendly, biocompatible, and economical. Other reports on the same have been able to show that melanin-based nanoparticle synthesis yields nanomaterials that have considerable antimicrobial and anticancer properties [3]. Importantly, the robustness of these outcomes was reinforced by statistical validation. Significant differences were consistently observed across experimental conditions (e.g., pH, temperature, nutrient sources, antimicrobial and antifungal activity, antioxidant assays, heavy-metal binding, and dye decolorization), confirming that the reported variations were not incidental but statistically meaningful.

Although it has shown encouraging outcomes, this study is also limited by the fact that it lacks in vivo validation, as well as molecular-level insights. The next steps should involve structural characterization of melanin with the help of such spectroscopic methods as FTIR, NMR, and mass spectrometry, as well as pharmacokinetic and toxicity studies in model organisms. The melanin produced by *Streptomyces chromofuscus* exhibits a broad functionality, such as antimicrobial, antioxidant, biosurfactant, bioremediation, and nanomaterial synthesis capabilities. The results make microbial melanin a potentially broad bioresource with extensive implications for pharmaceutical, environmental, and nanotechnological development.

## Conclusions

This research has shown that *S. chromofuscus* is a high-yielder of melanin with wide biological and technological applications. Starch was the best carbon source in stimulating melanin production among the tested carbon sources, followed by glycerol and fructose. Arginine, lysine, asparagine, cysteine, and proline, a source of nitrogen, also greatly promoted the production of pigments. The purified melanin was a potent antimicrobial against human and plant pathogens, and its antioxidant activity was confirmed by its capacity to prevent lipid peroxidation in linoleic acid systems for a minimum of six days, as opposed to 24 hours in the controls. Other than its biomedical significance, melanin of *S. chromofuscus* showed significant biosurfactant activity, as indicated by a distinct hydrolysis area on tributyrin agar, and may be applicable in emulsification and environmental uses. The pigment also demonstrated good chelation of such heavy metals as cadmium, cobalt, nickel, and zinc, which shows its application potential in bioremediation. Also, melanin was able to decolorize brilliant green azo dye by 97.95% and thus it is a dye degrader. It is important to note that it also promoted the biosynthesis of silver and copper nanoparticles, and copper sulfate produced the optimum formation of nanoparticles. Such multifunctional properties make microbial melanin an attractive candidate in terms of sustainable use in pharmaceuticals, nanotechnology, and environmental biotechnology. Nevertheless, additional structural determination and *in vivo* testing are suggested to promote its clinical application.
